# Outcomes of Mechanical Circulatory Support for Giant Cell Myocarditis: A Systematic Review

**DOI:** 10.3390/jcm9123905

**Published:** 2020-12-01

**Authors:** Preeyal M. Patel, Abhiraj Saxena, Chelsey T. Wood, Thomas J. O’Malley, Elizabeth J. Maynes, John W. C. Entwistle, H. Todd Massey, Preethi R. Pirlamarla, René J. Alvarez, Leslie T. Cooper, J. Eduardo Rame, Vakhtang Tchantchaleishvili

**Affiliations:** 1 Sidney Kimmel Medical College, Thomas Jefferson University, Philadelphia, PA 19107, USA; preeyalp@gmail.com (P.M.P.); a.s.abhiraj.saxena@gmail.com (A.S.); chelseytylerwood@gmail.com (C.T.W.); 2 Division of Cardiac Surgery, Department of Surgery, Thomas Jefferson University, Philadelphia, PA 19107, USA; tomalley89@gmail.com (T.J.O.); elizabethjanemd@gmail.com (E.J.M.); john.entwistle@jefferson.edu (J.W.C.E.); howard.massey@jefferson.edu (H.T.M.); eduardo.rame@jefferson.edu (J.E.R.); 3 Division of Cardiology, Department of Medicine, Thomas Jefferson University, Philadelphia, PA 19107, USA; preethi.pirlamarla@jefferson.edu (P.R.P.); rene.alvarez@jefferson.edu (R.J.A.); 4 Department of Cardiovascular Medicine, Mayo Clinic, Jacksonville, FL 32224, USA; cooper.leslie@mayo.edu

**Keywords:** myocarditis, mechanical circulatory support, immunosuppression, treatment, survival

## Abstract

Treatment of giant cell myocarditis (GCM) can require bridging to orthotopic heart transplantation (OHT) or recovery with mechanical circulatory support (MCS). Since the roles of MCS and immunotherapy are not well-defined in GCM, we sought to analyze outcomes of patients with GCM who required MCS. A systematic search was performed in June 2019 to identify all studies of biopsy-proven GCM requiring MCS after 2009. We identified 27 studies with 43 patients. Patient-level data were extracted for analysis. Median patient age was 45 (interquartile range (IQR): 32–57) years. 42.1% (16/38) were female. 34.9% (15/43) presented in acute heart failure. 20.9% (9/43) presented in cardiogenic shock. Biventricular (BiVAD) MCS was required in 76.7% (33/43) of cases. Of the 62.8% (27/43) of patients who received immunotherapy, 81.5% (22/27) used steroids combined with at least one other immunosuppressant. Cyclosporine was the most common non-steroidal agent, used in 40.7% (11/27) of regimens. Immunosuppression was initiated before MCS in 59.3% (16/27) of cases, after MCS in 29.6% (8/27), and not specified in 11.1% (3/27). Immunosuppression started prior to MCS was associated with significantly better survival than MCS alone (*p* = 0.006); 60.5% (26/43) of patients received bridge-to-transplant MCS; 39.5% (17/43) received bridge-to-recovery MCS; 58.5% (24/41) underwent OHT a median of 104 (58–255) days from diagnosis. GCM recurrence after OHT was reported in 8.3% (2/24) of transplanted cases. BiVAD predominates in mechanically supported patients with GCM. Survival and bridge to recovery appear better in patients on immunosuppression, especially if initiated before MCS.

## 1. Introduction

Giant cell myocarditis (GCM) is a rare autoimmune disorder characterized by diffuse myocardial necrosis with multinucleated giant cells [1]. First described in 1905 by Saltikow [2], GCM is often mistaken for other infiltrative cardiomyopathies [3] such as cardiac sarcoidosis and lymphocytic myocarditis. Although these diseases may present similarly and share some histopathologic features, GCM is most associated with a poor prognosis [4], resulting in severe heart failure and ventricular arrhythmias.

Given the rare incidence of GCM, there are limited data on the topic, with Cooper’s Multicenter Giant Cell Myocarditis Registry [5] as the largest collection of data in this population. GCM affects young individuals and is associated with autoimmune disease and tumors, especially thymoma. The mean age at the time of diagnosis is 52.5 years [6] for patients with GCM, with some presenting in young adulthood. Heart failure by contrast has a mean age of diagnosis at 75 years [7]. Since patients affected by giant cell myocarditis are younger than the average patient population experiencing heart failure, improved management guidelines may meaningfully impact their quality of life and long-term survival.

Most patients with GCM are treated with immunosuppression (IS) or orthotopic heart transplantation (OHT). With the development of durable, continuous-flow left ventricular assist devices (CF-LVADs), patients with end-stage heart failure can be mechanically supported as they await OHT [8]. When GCM presents as a fulminant myocarditis with acute heart failure, patients may be placed on bridge-to-transplant mechanical circulatory support (MCS) [9], and often, the diagnosis of GCM is made via biopsy at the time of LVAD implantation or transplant [10]. Given the rarity of GCM, there is limited literature discussing treatment outcomes in patients managed with MCS. The purpose of this study is to evaluate the characteristics and outcomes of immunosuppression in mechanically supported patients with giant cell myocarditis.

## 2. Experimental Section

### 2.1. Literature Search

**Figure 1:**
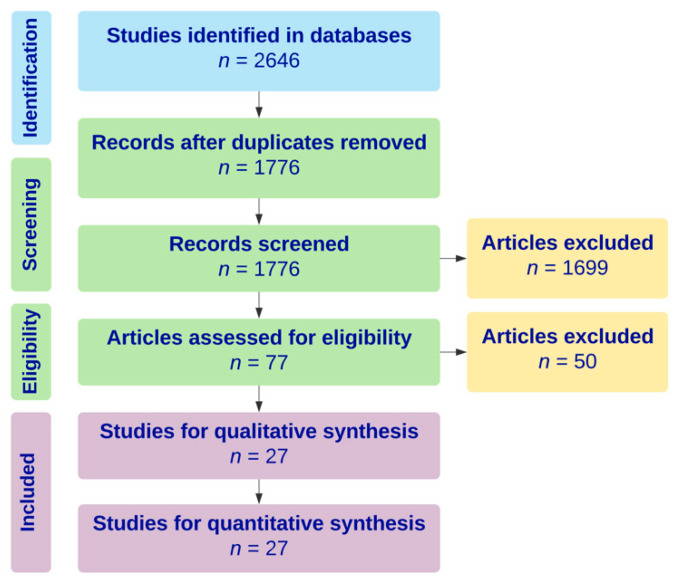
Preferred Reporting Items for Systematic Reviews and Meta-Analysis (PRISMA) diagram outlining literature search strategy for systematic review.

An electronic search was performed in June 2019 in the Cochrane, MEDLINE, Scopus, and Cumulative Index of Nursing and Allied Health Literature (CINAHL) databases to identify all reports of biopsy-proven giant cell myocarditis requiring mechanical circulatory support from 2009 through 2019. The keyword “giant cell myocarditis” was used to perform the search. Of the 2646 articles identified, 27 articles, consisting of six case series and 21 case reports, with a total of 43 cases met inclusion criteria. A Preferred Reporting Items for Systematic Reviews and Meta-Analysis (PRISMA) flow diagram outlining the literature search protocol is provided as Figure 1. No institutional review board approval was required for this study, given that all data were collected from previously published articles.

### 2.2. Inclusion Criteria

Only biopsy-proven cases of giant cell myocarditis were included in this study. All adults 18 years or older with MCS, including CF-LVADs, biventricular assist devices (BiVAD), and total artificial hearts (TAH), were included in the study. Due to limited available data, case series and case reports were selected.

### 2.3. Data Extraction

Patient-level data were extracted and pooled for statistical analysis from the body, figures, and tables of selected case series and case reports. In situations where data were not available, attempts were made to contact corresponding authors, but no additional data were successfully retrieved.

### 2.4. Statistical Analysis

Baseline characteristics were reported as descriptive statistics, including medians and interquartile ranges (IQR) for continuous variables and percentages for categorical variables. Patients were dichotomized into those who received MCS (MCS alone) and those who received immunosuppression in addition to MCS (MCS + IS). Individual patient survival and follow up data from each article were combined for Kaplan–Meier survival analysis censored for death. Log-rank test was performed to compare survival by timing of immunotherapy (IS pre-MCS, IS post-MCS, or MCS alone) and by MCS modality (LVAD or BiVAD). Additionally, immunosuppression status and use of temporary MCS before durable MCS as potential markers of patient acuity were examined using univariable and multivariable Cox proportional hazard regression models. *p*-values less than 0.05 were considered statistically significant. All analyses were performed with R software, version 3.5.1 (R Foundation for Statistical Computing, Vienna, Austria).

### 2.5. Evaluation of Missing Data

The articles included in this systematic review did not all report the same variables. The discrepancy required certain interpretations and adjustments to resolve the missing data. Denominators were adjusted when calculating percentage values for missing categorical data. Routine variables such as demographics and baseline characteristics were evaluated as missing at random, while variables representing major adverse events were interpreted to not have occurred if not explicitly mentioned. Given that the missing baseline characteristics were not necessary for survival analysis, we determined that the missing data would not meaningfully impact our discussion of survival outcomes, the primary focus of this paper. Therefore, no further intervention beyond denominator adjustment was performed.

## 3. Results

### 3.1. Baseline Characteristics

Median patient age was 45 (IQR: 32–57) years, and 42.1% (16/38) of patients were female; 20.0% (8/40) of patients had a history of autoimmune disease, including 7.5% (3/40) with Hashimoto’s thyroiditis, 5.0% (2/40) with rheumatoid arthritis, 2.5% (1/40) with celiac disease, 2.5% (1/40) with Grave’s disease, and 2.5% (1/40) with ulcerative colitis. No patients reported a history of thymoma or other tumors. Baseline characteristics of patients included in the study are shown in Table 1.

Patients reported the onset of symptoms a median of 7 (7–10) days prior to presentation. 34.9% (15/43) of patients presented in acute heart failure, and 20.9% (9/43) presented in cardiogenic shock. Arrhythmias were detected in 57.1% (24/42) of patients, with complete atrioventricular (AV) block and ventricular tachycardia being the most common. Of note, patients who received MCS + IS had a significantly higher left ventricular ejection fraction (LVEF) at presentation compared to patients who received MCS alone (28% (20–30) vs. 17% (13–20), *p* = 0.03). The final diagnosis of GCM was made using endomyocardial biopsy in 72.1% (31/43) of cases, left ventricular apical core biopsy in 23.3% (10/43) of cases, and biopsy of the explanted heart in 2.3% (1/43) of cases.

Patients in both groups were similarly supported with temporary MCS prior to LVAD implantation. These temporary devices included veno-arterial extracorporeal membrane oxygenation (VA ECMO) in 25.0% (9/36) of cases, intra-aortic balloon pump (IABP) in 16.7% (6/36) of cases, and Impella in 7.0% (3/43) of cases. Two patients (4.7%) were transitioned from temporary LVAD to durable LVAD support, and five (11.6%) patients were transitioned from temporary LVAD to durable BiVAD support. In two cases, a temporary right ventricular assist device (RVAD) was placed before transitioning to a BiVAD.

The majority of patients (76.7%, 33/43) required a BiVAD, and the remaining 23.3% (10/43) of patients received an LVAD. Most patients were mechanically supported as bridge-to-transplant (60.5%, 26/43), while the remainder were supported as bridge-to-recovery (39.5%, 17/43). Patients were maintained on MCS for a median of 39 (15–137) days. There was no significant difference in MCS characteristics between the MCS alone and MCS + IS subgroups. A detailed outline of the temporary and durable types of MCS used are depicted in Table 2.

An immunosuppressive regimen was used in 62.8% (27/43) of cases. Immunosuppression was initiated before MCS (IS pre-MCS) in 59.3% (16/27) of cases, after MCS (IS post-MCS) in 29.6% (8/27) of cases, and not specified in 11.1% (3/27) of cases. Of the patients receiving MCS + IS, the vast majority, 81.5% (22/27), received combined immunosuppression consisting of steroids in combination with at least one other immunosuppressant. Cyclosporine was the most common, administered to 40.7% (11/27) of patients. Other immunosuppressants included in the regimens are listed in Table 3. Among patients requiring maintenance immunosuppression to prevent recurrence, steroids were the most common long-term therapy, used in 42.3% (11/26) of cases.

### 3.2. Complications and Outcomes

Complications and outcomes of MCS and immunotherapy are detailed in Table 4. At least one infectious adverse event was reported in 50.0% (6/12) of cases, three of which were LVAD infections; 18.2% (2/11) of the patients on maintenance immunosuppression were discontinued from immunosuppressive therapy due to LVAD infection. Five patients received devices for management of persistent arrhythmias, including a dual chamber pacemaker in 9.3% (4/43) of patients and implantable cardioverter defibrillator in one patient (5%, 1/21). Recurrence of GCM after OHT was reported in 8.3% (2/24) of transplanted cases.

**Figure 2:**
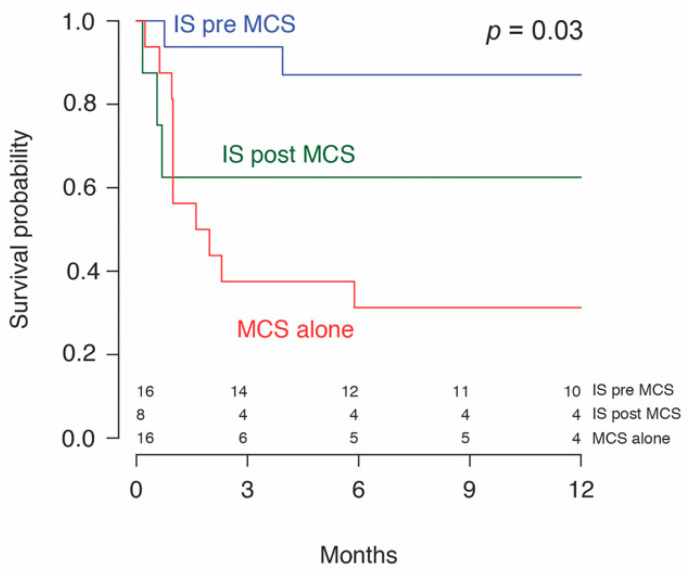
Survival probability of patients with giant cell myocarditis treated with immunosuppression (IS) before or after mechanical circulatory support (MCS).

**Figure 3:**
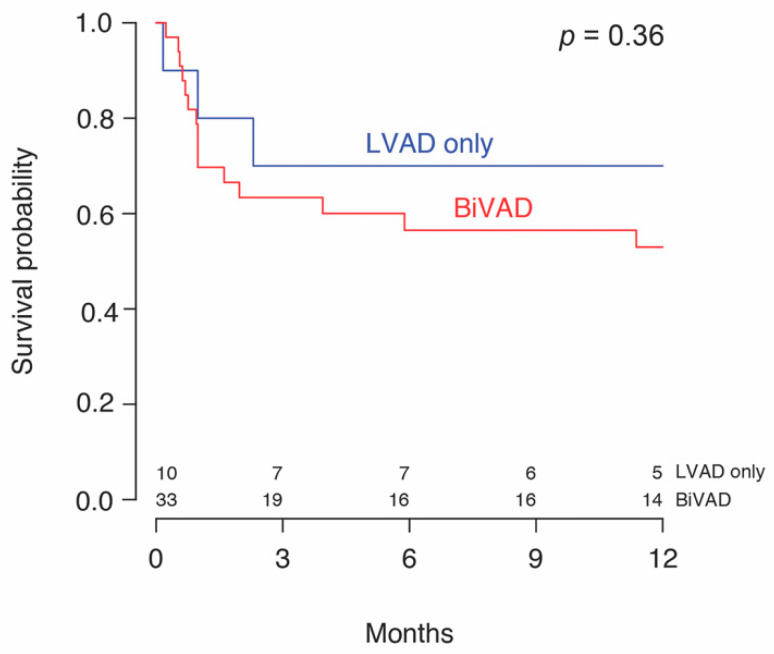
Survival probability of patients with giant cell myocarditis by left ventricular support (LVAD) alone or biventricular (BiVAD) support.

One patient in the MCS alone subgroup was successfully weaned of LVAD support on day 222. No other patients achieved a recovery on MCS as these patients were either transplanted or lost to follow-up. Two patients (2/43, 4.7%) remained on MCS at the end of the follow-up. There were no deaths in the bridge to recovery group. 58.5% (24/41) underwent OHT within a median time of 46 (18–201) days from initiation of MCS. A median transplant-free survival of 104 (58–255) days or 3.5 months from diagnosis to transplant was found, with similar values in both groups. No significant difference in 30-day mortality, combined 30.2% (13/43), or overall mortality at the end of follow-up, combined 51.2% (22/43), was detected between the two groups. Patients receiving MCS + IS had a significantly greater 1-year survival (72.7% (16/22) vs. 31.2% (5/16), *p* = 0.03) compared to patients on MCS alone. Kaplan–Meier survival analysis stratified by immunosuppression (Figure 2) and mode of MCS (Figure 3), is shown. Patients with LVADs and patients with BiVADs had similar survival on Kaplan–Meier analysis (*p* = 0.36). Table 5 illustrates how immunosuppression status and use of temporary MCS before durable MCS relate to mortality, by summarizing the results of univariable and multivariable analysis. Neither immunosuppression nor initial use of temporary MCS were found to be significantly associated with mortality on multivariable analysis. However, the hazard ratio of immunosuppression was significantly low (*p* = 0.049) in univariable analysis and approaching significance (*p* = 0.078) in multivariable analysis. Regarding the timing of initiation of immunotherapy, immunosuppressive treatment started prior to MCS (IS pre-MCS) initiation was associated with significantly better survival than MCS alone (*p* = 0.006). We found no difference in survival between the IS post-MCS and MCS only subgroups and the IS post-MCS and IS pre-MCS subgroups.

## 4. Discussion

### 4.1. Discussion of Clinical Significance

This study adds to the available literature on the use of MCS for GCM by describing the relative rates of recovery, transplant, and infection in a cohort of patients receiving contemporary modalities of mechanical support and immunosuppression. Our study highlights the effectiveness of timely immunosuppression in prolonging survival in this sick population.

Currently, treatment options for GCM include immunosuppression or, in severe cases, OHT, although patients often die awaiting transplant. Even after OHT, spontaneous recurrence of disease may occur, first noted [1] by Kong et al. [11] and then Gries et al. [12]. While Gries et al. [12]. recommend a more cautious transplant evaluation, Kong et al. [11] and Cooper et al. [13] recommend transplant and triple-immunosuppression. The effectiveness of immunosuppression has been previously demonstrated in a 1997 study which found immunosuppression increased median transplant-free survival in patients with GCM from three to 12.3 months [13] after diagnosis. More recently, Maleszewski et al. [14] reported the possibility of surviving over 19 years [14] on contemporary immunosuppression alone without the need for transplant. When detected early, typically in young patients who undergo further testing after discovery of an unexplained distal AV block, immunotherapy achieves a modest clinical remission in two thirds of patients [6]. Patients with fulminant GCM experience different outcomes from those with a milder progression of disease [15]. These patients with rapidly progressive heart failure likely require the institution of MCS to mitigate the effects of GCM on hemodynamic stability and end-organ function which may not be sufficiently treated by immunosuppression alone [14].

There are a variety of temporary and durable options for MCS in the management of GCM. Of the temporary options, VA ECMO has been considered favorably [16] in the initial management of patients with GCM presenting in cardiogenic shock given its ability to provide temporary stabilization until a diagnosis is made or the need for durable MCS is determined [17]. With VA ECMO support, there is a need to unload the ventricle with venting or Impella, which has been shown to have disease-modifying effects [18,19]. CF-LVADs and BiVADs, through improved quality of life, organ performance, and hemodynamic stability, have extended the life expectancy of patients with end-stage heart failure [20,21]. Since patients with GCM have a similar post-transplant survival [22] as patients with all other heart diseases treated with OHT, bridging patients to transplant is crucial and valuable. Another reported benefit [16] of MCS is its ability to support the ventricle if it becomes irritated after EMB, the gold-standard [23] for GCM diagnosis. Early biopsy is critical for patients with suspected GCM, given that rates of transplant and death have been reported [17] as high as 80% to 100% in patients who do not receive a biopsy. Patients often require repetitive biopsies when the results are inconclusive or disease recurrence is suspected [24]. MCS can relieve the stress on the ventricle following EMB, thus preventing exacerbation of pre-existing arrhythmias [25], a known complication of EMB [26].

Regarding durable MCS options for GCM, biventricular support is recommended given that the inflammation of GCM can affect both ventricles [27]. However, we did not detect a difference in survival between patients on CF-LVADs and those on BiVADs. The majority of patients in our study received a BiVAD, and the decision to use a CF-LVAD in place of BiVAD in 23.3% (10/43) of patients was not specified. In three of the patients receiving a CF-LVAD, GCM was diagnosed after device implantation. In these cases, patients likely presented in a severe condition requiring rapid MCS before a diagnosis could be made. Additionally, BiVADs are used in patients with a concomitant right ventricular failure who are unlikely to recover. Patients who were predicted to recover likely received a bridge-to-recovery LVAD instead of a BiVAD. One patient in our study was supported with TAH. Although there are limited data on the use of TAH in GCM, one study reports [20] a good prognosis and recommends its use as a bridge to transplant when a longer time to transplant is expected, such as in patients with blood types O and A.

When examining the patient subgroups with improved survival, treatment involving both immunosuppression and MCS (MCS + IS) was associated with better overall survival compared to treatment with MCS alone. Immunosuppression may reduce the severity of heart failure in these patients by preventing further autoimmune destruction of the myocardium. These patients may also have had better survival because they presented in less severe heart failure. At the time of initial presentation, we observed that patients who received MCS + IS had a significantly higher LVEF at presentation compared to patients who received MCS alone (28% (20–30) vs. 17% (13–20), *p* = 0.03). Previous studies [17] have also reported that patients with a milder presentation or insidious course have less severe outcomes than those who present in acute cardiac compromise. Specifically in this study, the administration of immunosuppression prior to MCS implantation (IS pre-MCS) was associated with increased survival. However, given the limitations of these data, it is not clear why IS pre-MCS is effective. The ability to initiate IS prior to MCS may indicate a more indolent disease course, allowing more time for IS to be instituted when compared to acutely severe presentations requiring rapid MCS intervention. Since we observed that patients with a higher systolic function at presentation tended to receive IS more frequently and survived longer, future studies investigating LVEF as a factor in determining candidates eligible for early immunosuppression may be valuable.

Our study introduces a key element in clinical management of patients with GCM: timing of immunosuppression. Early biopsy and immunosuppression have been recommended [16], but there are few data describing its exact impact on survival. To our knowledge, our study is the first to provide evidence that early immunosuppression (defined as the initiation of IS prior to MCS) is associated with better long-term survival. Immunosuppression likely reduces the severity of heart failure by curbing the autoimmune destruction of the myocardium. Patients receiving IS pre-MCS may also have had a milder disease progression, allowing more time for institution of IS prior to MCS.

### 4.2. Limitations and Future Directions

Since we extracted data from articles with different purposes, we were unable to collect all variables for each patient. Those receiving MCS alone could be sicker than patients receiving MCS + IS, as suggested by the difference in LVEF at presentation between these two groups. However, given the lack of granular data, we could not further characterize the severity of illness or control for confounding effects. Additionally, our study included all immunosuppressants under a broad “immunosuppression” category, so we are not able to provide more detail on the relative effectiveness of specific drugs. During the 10-year period encompassed by this study, immunosuppression has evolved. Triple immunosuppression [28] with corticosteroids, cyclosporine, and azathioprine or mycophenolate mofetil has been widely used in GCM, along with antithymocyte globulin in the modern era. Clinical outcomes may vary by regimen.

It is important to consider infection in patients who are immunosuppressed. One study reported that 75% of patients on long-term immunosuppression for GCM experienced at least one infection-related readmission [29]. Only 12 cases in our study mentioned infection. Infection was likely underreported, and we felt it was inappropriate to assume the absence of infection when it was not explicitly stated. Future investigations into infectious complications may be valuable in guiding clinical management.

## 5. Conclusions

Most patients with GCM who require MCS need BiVAD support. Survival in patients with GCM requiring MCS may be improved when immunosuppression is initiated before MCS implantation.

## Figures and Tables

**Table 1:** Baseline characteristics of patients with giant cell myocarditis (GCM) supported with mechanical circulatory support (MCS) alone or in combination with immunosuppression.

Baseline Characteristics ^1^	MCS Alone (*n* = 16)	MCS + IS (*n* = 27)	Total (*n* = 43)	*p*-Value
Female	5/11 (45.5)	11/27 (40.7)	16/38 (42.1)	1.00
Age at diagnosis (years)	41.0 (30.0, 55.0)	49.0 (34.5, 58.5)	44.5 (32.2, 56.5)	0.25
History of autoimmune disease	1/16 (6.2)	7/24 (29.2)	8/40 (20.0)	0.17
History of thymoma or other tumors	0/16 (0.0)	0/24 (0.0)	0/40 (0.0)	1.00
Clinical presentation				
Acute heart failure	5/16 (31.2)	10/27 (37.0)	15/43 (34.9)	0.96
Shortness of breath	6/16 (37.5)	4/27 (14.8)	10/43 (23.3)	0.18
Cardiogenic shock	3/16 (18.8)	6/27 (22.2)	9/43 (20.9)	1.00
Presyncope	3/16 (18.8)	3/27 (11.1)	6/43 (14.0)	0.81
Chest pain	2/16 (12.5)	2/27 (7.4)	4/43 (9.3)	0.99
Cardiac arrest requiring CPR	0/16 (0.0)	1/27 (3.7)	1/43 (2.3)	1.00
ST segment elevation	1/16 (6.2)	4/27 (14.8)	5/43 (11.6)	0.72
Arrhythmia	11/15 (73.3)	13/27 (48.1)	24/42 (57.1)	0.21
Ventricular tachycardia	5/15 (33.3)	7/25 (28.0)	12/40 (30.0)	1.00
Complete AV block	3/15 (20.0)	4/25 (16.0)	7/40 (17.5)	1.00
Right bundle branch block	2/15 (13.3)	0/22 (0.0)	2/37 (5.4)	0.31
Atrial fibrillation	0/15 (0.0)	1/25 (4.0)	1/40 (2.5)	1.00
Time from symptoms to admission (days)	7 (6, 7)	8 (7, 11)	7 (7, 10)	0.20
Elevated cardiac enzymes	8/16 (50.0)	3/27 (11.1)	11/43 (25.6)	0.13
LVEF at presentation (%)	17.0 (12.5, 20.0)	28.0 (20.0, 30.0)	20.0 (15.0, 28.2)	0.03
Biopsy proven	16/16 (100.0)	27/27 (100.0)	43/43 (100.0)	0.09
Endomyocardial biopsy	9/16 (56.2)	22/27 (81.5)	31/43 (72.1)	0.15
Apical sample during LVAD implantation	6/16 (37.5)	4/27 (14.8)	10/43 (23.3)	0.18
Explanted heart	1/43 (6.2)	0/43 (0.0)	1/43 (2.3)	0.79
Time from symptoms to diagnosis (days)	16 (4,21)	21 (9,54)	18.5 (7,54)	0.51
Initial inotropic support	11/14 (78.6)	13/25 (52.0)	24/39 (61.5)	0.20

^1^ All values are reported as *n* (%) or median (interquartile range (IQR)). Abbreviations—MCS: mechanical circulatory support, IS: immunosuppression, CPR: cardiopulmonary resuscitation, AV: atrioventricular, LVEF: left ventricular ejection fraction, LVAD: left ventricular assist device.

**Table 2:** MCS characteristics in patients with GCM.

MCS Characteristics ^1^	MCS Only (*n* = 16)	MCS + IS (*n* = 27)	Total (*n* = 43)	*p*-Value
MCS as initial treatment	16/16 (100.0)	16/19 (84.2)	32/35 (91.4)	0.73
MCS after immunosuppression failed		3/19 (15.8)	3/35 (8.6)	
Time from admission to MCS (days)	14 (5, 39)	8 (4, 17)	9 (4, 22)	0.38
Intra-aortic balloon pump	2/14 (14.3)	4/22 (18.2)	6/36 (16.7)	1.00
Impella	0/16 (0.0)	3/27 (11.1)	3/43 (7.0)	0.45
VA ECMO	2/13 (15.4)	7/23 (30.4)	9/36 (25.0)	0.55
Temporary LVAD prior to durable LVAD	1/16 (6.2)	1/27 (3.7)	2/43 (4.7)	1.00
Temporary LVAD prior to durable BiVAD	3/16 (18.8)	2/27 (7.4)	5/43 (11.6)	0.53
Any LVAD	16/16 (100.0)	27/27 (100.0)	43/43 (100.0)	0.09
Bridge to transplant	8/16 (50.0)	18/27 (66.7)	26/43 (60.5)	0.45
Bridge to recovery	8/16 (50.0)	9/27 (33.3)	17/43 (39.5)	0.45
Time from admission to LVAD (days)	14 (5, 39)	8 (4, 17)	9 (4, 22)	0.38
VAD support duration (days)	20 (13, 98)	48 (17, 137)	39 (15, 137)	0.54
Durable LVAD	4/16 (25.0)	6/27 (22.2)	10/43 (23.3)	1.00
HeartMate II LVAD	3/16 (18.8)	2/27 (7.4)	5/43 (11.6)	0.53
Jarvik 2000 LVAD	1/16 (6.2)	1/27 (3.7)	2/43 (4.7)	1.00
Unspecified durable LVAD	0/16 (0.0)	3/27 (11.1)	3/43 (7.0)	0.45
Any biventricular support	12/16 (75.0)	21/27 (77.8)	33/43 (76.7)	1.00
Durable BiVAD	4/16 (25.0)	7/27 (25.9)	11/43 (25.5)	0.95
HeartWare BiVAD	1/16 (6.2)	3/27 (11.1)	4/43 (9.3)	1.00
Total artificial heart	0/16 (0.0)	1/27 (3.7)	1/43 (2.3)	1.00
Unspecified durable BiVAD	3/16 (18.8)	1/27 (3.7)	4/43 (9.3)	1.00
Durable LVAD + Temporary RVAD	1/16 (6.2)	2/27 (7.4)	3/43 (7.0)	0.88
HeartMate II LVAD + Centrimag RVAD	1/16 (6.2)	1/27 (3.7)	2/43 (4.7)	1.00
HeartMate II LVAD + Abiomed RVAD	0/16 (0.0)	1/27 (3.7)	1/43 (2.3)	1.00
Temporary Centrimag BiVAD	4/16 (25.0)	4/27 (14.8)	8/43 (18.6)	0.67
Unspecified BiVAD	3/16 (18.8)	8/27 (29.6)	11/43 (25.5)	0.43

^1^ All values are reported as *n* (%) or median (IQR). Abbreviations—MCS: mechanical circulatory support, GCM: giant cell myocarditis, IS: immunosuppression, VA ECMO: veno-arterial extracorporeal membrane oxygenation, LVAD: left ventricular assist device, BiVAD: biventricular assist device, RVAD: right ventricular assist device.

**Table 3:** Characteristics of immunotherapy associated with short and long-term maintenance.

Characteristics of Immunotherapy ^1^	MCS + IS (*n* = 27)
**Initial (short-term) immunosuppression**	
Immunosuppressants per regimen	2.0 (2.0, 2.0)
Immunosuppression as first-line treatment	16/24 (66.7)
Immunosuppression started after VAD	8/24 (33.3)
Steroid-free regimen	1/27 (3.7)
Steroids alone	4/27 (14.8)
Combined steroids + other immunosuppressant ^2^	22/27 (81.5)
Cyclosporine	11/27 (40.7)
Cyclophosphamide	5/27 (18.5)
IV immunoglobulin	4/27 (14.8)
Antithymocyte globulin	2/27 (7.7)
Mycophenolate mofetil	2/27 (7.4)
Azathioprine	1/27 (3.7)
Tacrolimus	1/27 (3.7)
Rituximab	1/27 (3.8)
Muromonab	1/27 (3.7)
**Maintenance (long-term) immunosuppression**	
Maintenance immunosuppression ^2^	11/27 (40.7)
Steroids	11/26 (42.3)
Cyclosporine	2/26 (7.4)
Mycophenolate mofetil	6/26 (23.1)
Tacrolimus	5/26 (19.2)
Rituximab	1/26 (3.8)

^1^ All values are reported as *n* (%) or median (IQR). ^2^ Immunosuppressants total to >100% as some patients had more than one agent in regimen. Abbreviations—MCS: mechanical circulatory support, IS: immunosuppression, VAD: ventricular assist device, IV: intravenous.

**Table 4:** Complications and outcomes of patients with GCM treated with MCS alone or in combination with immunosuppression.

Complications and Outcomes ^1^	MCS alone (*n* = 16)	MCS + IS (*n* = 27)	Total (*n* = 43)	*p*-Value
Complications				
At least one infectious adverse event	1/2 (50.0)	5/10 (50.0)	6/12 (50.0)	1.00
Recurrent GCM	2/16 (12.5)	3/27 (11.1)	5/43(11.6)	1.00
Recurrent GCM after OHT	0/16 (0.0)	2/27 (7.4)	2/43 (4.7)	0.71
New dual-chamber pacemaker placed	1/16 (6.3)	3/27 (1.1)	4/43 (9.3)	0.34
Renal failure	1/16 (6.3)	1/19 (5.3)	2/35 (5.7)	0.90
Severe hemorrhage	1/1 (100.0)	1/3 (33.3)	2/4 (50.0)	1.00
Outcomes				
Transplant	7/15 (46.7)	17/26 (65.38)	24/41 (58.5)	0.24
Time from diagnosis to transplant (days)	175 (123, 270)	70 (48, 247)	104 (58, 255)	0.35
Overall mortality at the end of follow-up	11/16 (68.8)	11/27 (40.7)	22/43 (51.2)	0.14
30-day mortality	8/16 (50.0)	5/27 (18.5)	13/43 (30.2)	0.07
One-year survival	5/16 (31.2)	16/22 (72.7)	21/38 (55.3)	0.03
Duration of follow up (months)	1.8 (1.0, 14.0)	24.0 (3.5, 36.2)	11.5 (1.0, 34.5)	0.06

**^1^** All values are reported as *n* (%) or median (IQR). Abbreviations—MCS: mechanical circulatory support, IS: immunosuppression, GCM: giant cell myocarditis, OHT: orthotopic heart transplantation, LVAD: left ventricular assist device.

**Table 5:** Univariable and multivariable analysis for overall mortality.

	Univariable		Multivariable	
Variables	HR (95% CI)	*p*-Value	HR (95% CI)	*p*-Value
Immunosuppression used	0.42 (0.18–0.99)	0.049	0.45 (0.19–1.10)	0.078
tMCS * used before durable MCS	0.58 (0.23–1.45)	0.246	0.68 (0.27–1.75)	0.429

* Includes VA-ECMO, IABP, Impella, and temporary VAD, compared to use of durable MCS alone. Abbreviations—HR: hazard ratio, CI: confidence interval, tMCS: temporary MCS, MCS: mechanical circulatory support, VA-ECMO: venoarterial extracorporeal membrane oxygenation, IABP: intra-aortic balloon pump, VAD: ventricular assist device.
